# Panoramic dental tomosynthesis imaging by use of CBCT projection data

**DOI:** 10.1038/s41598-023-35805-1

**Published:** 2023-05-31

**Authors:** Taejin Kwon, Da-in Choi, Jaehong Hwang, Taewon Lee, Inje Lee, Seungryong Cho

**Affiliations:** 1 Department of Nuclear and Quantum Engineering (NQE), Korea Advanced Institute of Science and Technology, Daejeon, 34141 Korea; 2 Department of ICT, Dentium Co., Ltd., Suwon, Korea; 3 KAIST Institutes for ITC and HST, Korea Advanced Institute of Science and Technology, Daejeon, 34141 Korea

**Keywords:** Cone-beam computed tomography, Digital radiography in dentistry, Panoramic radiography

## Abstract

Dental CBCT and panoramic images are important imaging modalities used in dental diagnosis and treatment planning. In order to acquire a panoramic image without an additional panoramic scan, in this study, we proposed a method of reconstructing a panoramic image by extracting panoramic projection data from dental CBCT projection data. After specifying the patient’s dental arch from the patient’s CBCT image, panoramic projection data are extracted from the CBCT projection data along the appropriate panoramic scan trajectory that fits the dental arch. A total of 40 clinical human datasets and one head phantom dataset were used to test the proposed method. The clinical human dataset used in this study includes cases in which it is difficult to reconstruct panoramic images from CBCT images, such as data with severe metal artifacts or data without teeth. As a result of applying the panoramic image reconstruction method proposed in this study, we were able to successfully acquire panoramic images from the CBCT projection data of various patients. The proposed method acquires a universally applicable panoramic image that is less affected by CBCT image quality and metal artifacts by extracting panoramic projection data from dental CBCT data and reconstructing a panoramic image.

## Introduction

In dentistry, various advanced digital technologies such as optical imaging, 2D/3D x-ray imaging, and 3D printing are actively investigated and deployed^1–6^. In particular, dental CBCT and panoramic imaging are daily used imaging modalities in dental diagnosis and treatment planning^1,7^. Panoramic radiography plays an important role in the diagnosis of various dental diseases and their related treatment planning for it can provide relatively rich dental information in a single display with a wide field of view at a low imaging radiation dose^8–12^. The degree of dose may differ depending on the model and the scanning method, but in general, a panoramic radiograph has an effective dose from 8 to 14 µSv while that of a dental CBCT ranges from 10 to 130 µSv^13,14^. At the cost of a higher dose of CBCT, its ability to display anatomical information in 3D is considered crucial for diagnosis and therapy planning in various fields such as implant planning, abnormal teeth visualization, and jaw evaluation^7,15–17^. The visualization of dental CBCT image is usually provided with a dynamic volume rendered-view function and also with multiplanar reconstruction (MPR) slices. However, since panoramic image can comprehensively show anatomical structures with a wide FOV in a single plane, the demands for panoramic imaging are high even after CBCT image acquisition in clinics^18–26^. We would like to note that we do not aim to propose replacing dental panoramic imaging for initial diagnosis with CBCT imaging in this work but instead propose a method for synthesizing panoramic imaging once CBCT imaging has taken place and additional panoramic imaging is required thereafter. Having two separate systems is not desirable in terms of cost and space requirement and there exist imaging devices that provide both imaging modalities on a single CBCT platform.

There are two main approaches to acquiring dental CBCT and panoramic images from a single device: hardware and software approaches. In the hardware approach, the panoramic scan is available with CBCT equipment by allowing the rotating shaft of the CBCT equipment to move and implementing a narrow beam geometry by use of a collimator. The projection data are acquired along the predefined panoramic scan trajectory in the panoramic scan system. However, due to the fixed scan trajectory, out-of-focus panoramic images may be obtained when the patient’s dentition is substantially off the focal plane. More importantly, an additional panoramic scan would result in an additional imaging radiation dose to the patient.

In the software approaches, a panoramic image is fabricated from the dental CBCT image^18–21^. Since a dental CBCT image has 3D anatomical information, it is possible to produce a panoramic image by extracting necessary data from a CBCT image. In general, methods for synthesizing a panoramic image from a dental CBCT image consist of two steps^18^. The first step is to extract an appropriate dental arch that represents the teeth and jawbone from the dental CBCT image. The second step is to superpose the volumetric image information near the extracted dental arch into a panoramic image plane. Thus, a panorama image can be acquired without additional hardware changes to the existing dental CBCT equipment. In addition, unlike the panoramic scan system, there is no out-of-focus problem because the panoramic image is extracted from the CBCT image along the dental arch. However, these methods are greatly affected by the quality of the CBCT image and the accuracy of the dental arch^18–21,23–30^. Several algorithms have been reported to automatically extract sophisticated dental arches, but the accuracy of the dental arch is degraded when there are missing teeth and/or metal artifacts in the CBCT image^18,24^. In particular, when metal artifacts are severe, even if the ideal dental arch is set, metal artifacts of CBCT remain in the panoramic image, reducing the quality of the panoramic image.

To overcome the limitations of such software approaches, in this paper, we propose a novel panoramic image formation method that makes use of the CBCT projection data. Since the CBCT scan provides projection data of the FOV in the full angular range, it is in theory possible to selectively extract and reform the panoramic projection data from the CBCT projection data. In practice, an offset detector geometry is often used to cover a larger FOV in dental CBCT where the flat-panel detector center is horizontally placed off the principal x-ray of the system. Although there exists a sampling deficiency issue, as a result of the offset detector geometry, in the conversion process of cone-beam projection to panoramic projection data, we have devised a method to get around this problem as will be detailed in the “Materials and method” section. For extracting panoramic projection data from CBCT projection data, an appropriate dental arch is first delineated from a CBCT image^18,23^. A virtual panoramic scan trajectory suitable for the detected dental arch is then determined. At each source position along the virtual panoramic scan trajectory, panoramic projection data is extracted from the CBCT projection data through a data rebinning process. Finally, the panoramic tomosynthesis reconstruction method is applied to obtain a panoramic image from the extracted panoramic projection data^4,12,22^. Compared to the hardware approach, the proposed panoramic image reconstruction method has the advantage of obtaining a panoramic image at the focal plane optimized for a patient since the dental arch can be set patient-specifically from the CBCT image. Compared to the software approach that uses dental CBCT image instead of projection data, the proposed method is advantageous because it is not dominated by the quality of the CBCT image since it reconstructs the panoramic image directly from the extracted projection data.

The remainder of this paper is structured as follows. In section “Materials and method”, the overall structure of the proposed algorithm is given and details of each module are described. In section “Results”, experimental results in a head phantom scan data and clinical dataset are provided demonstrating the aforementioned advantages over the existing methods. Discussion and conclusions are followed in sections “Discussion” and “Conclusions”, respectively.

## Materials and method

**Figure 1:**
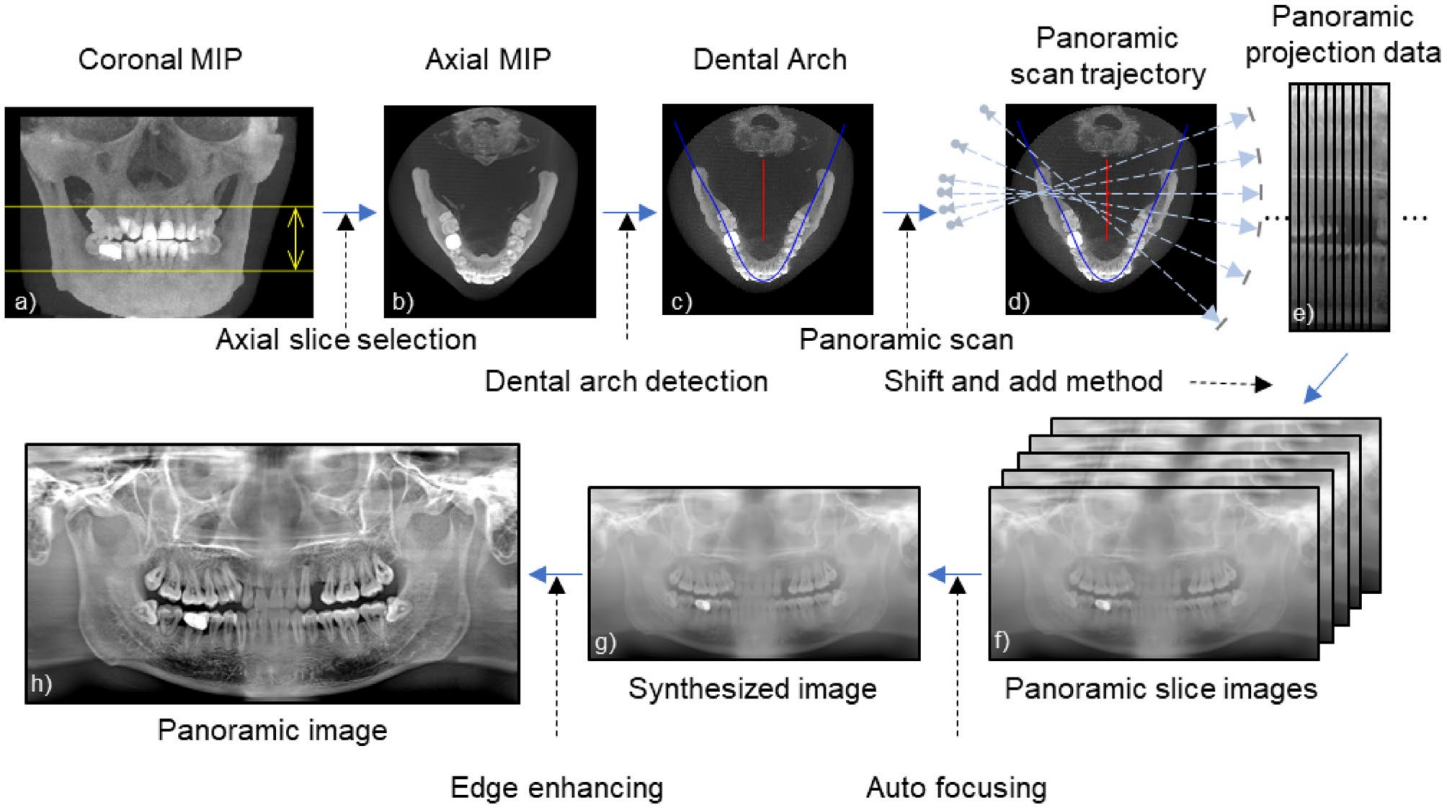
Workflow of the proposed method. (MIP stands for the maximum intensity projection).

An overview of the proposed method is schematically shown in Fig. 1. The first step is to set a dental arch suitable for the patient in the CBCT image (Fig. 1c). The next step is to establish a panoramic scan trajectory based on the set dental arch (Fig. 1d) and acquire virtual panoramic projection data from the CBCT projection data (Fig. 1e). Finally, a panoramic image is reconstructed based on the acquired panoramic projection data (Fig. 1h).

### Dental arch detection

An algorithm that automatically detects the dental arch from a CBCT image is described in this section. We use the axial maximum intensity projection (MIP) of the CBCT image that can represent the overall structure of the teeth^18,23–30^. An MIP image of the axial slices within a specified range is desirable for detecting dental arch. To distinguish the jawbone from the teeth in the axial MIP image, the range for generating the axial MIP image must be appropriately chosen^18^. If the slice range is set too wide, excessive bone tissue is superimposed on the axial MIP image, complicating the detection of the dental arch. On the other hand, there is a possibility that the region of interest may not be included when the slice range is set too narrow. In this work, we automatically determined the slice range using a coronal MIP image.

**Figure 2:**
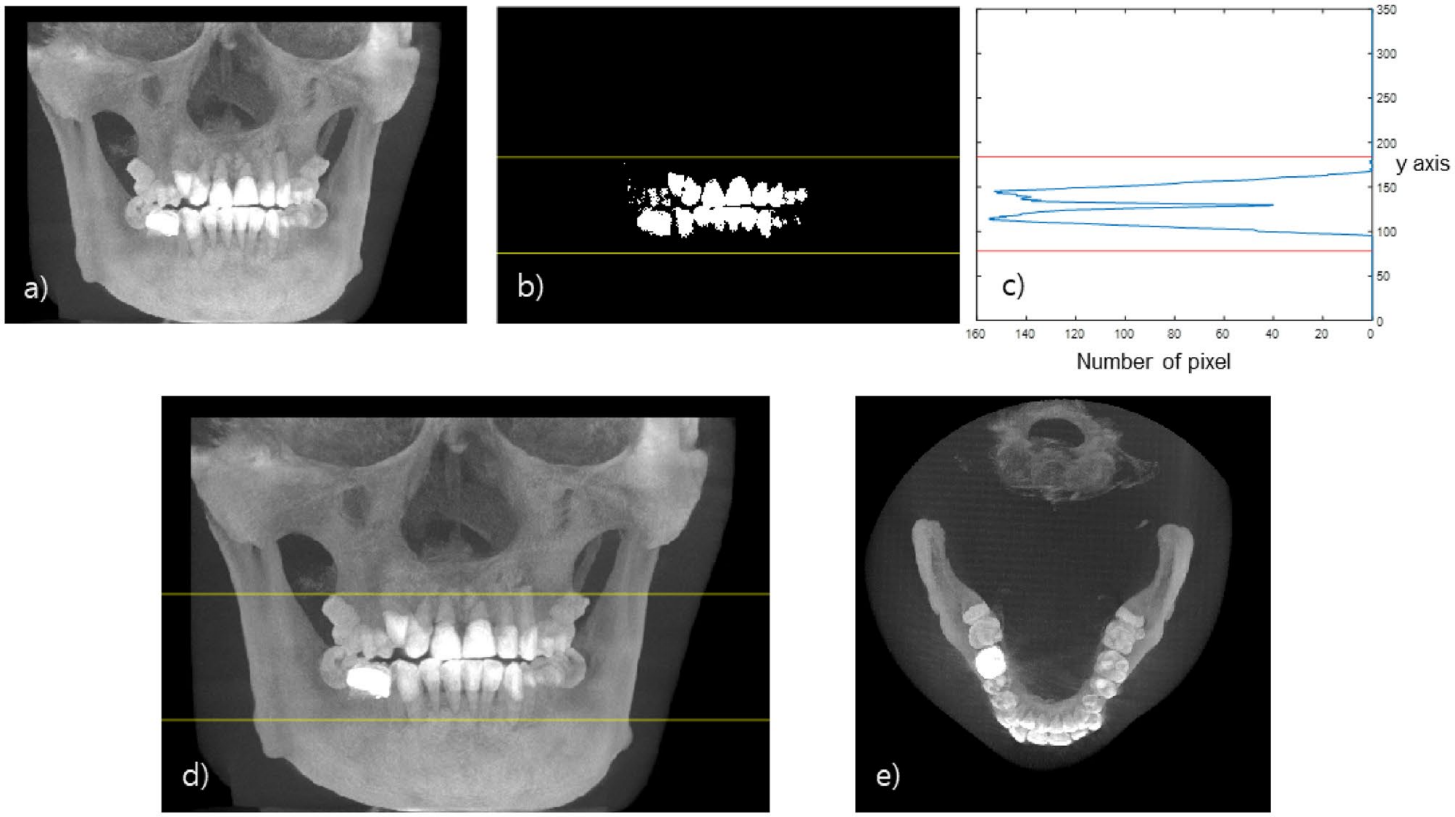
Range of the axial slice containing the teeth. (**a**) Coronal MIP image, (**b**) Mask image of teeth. (**c**) Y-axis histogram of the tooth mask image, (**d**) Selected range of the axial slice, (**e**) Axial MIP image.

First, a teeth binary mask (Fig. 2b) is acquired by applying a threshold, that has been empirically determined from the clinical dataset, to the coronal MIP image (Fig. 2a). A histogram that plots the number of positive pixels in each row of the mask image is prepared (Fig. 2c). From the histogram, we define the upper and lower bounds of the teeth in the row axis by thresholding the counts. Next, the axial slice range is set by allowing an additional margin of 20 slices which was also empirically determined in this work (Fig. 2d). Finally, an axial MIP image is obtained from the original CBCT image using the selected axial range (Fig. 2e).

**Figure 3:**
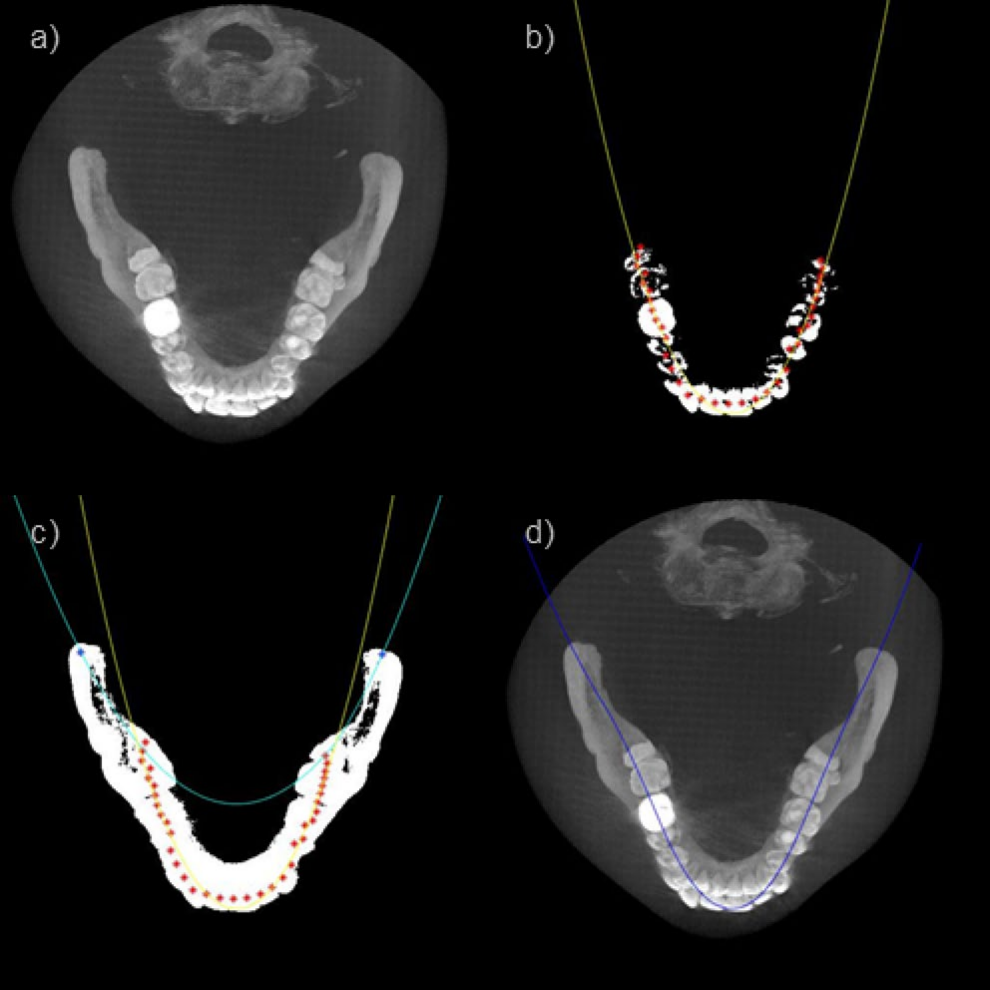
(**a**) Axial MIP image, (**b**) Tooth trajectory fitting, (**c**) Jaw bone trajectory fitting, (**d**) Dental arch detection.

After acquiring the appropriate axial MIP image, we detect the dental arch based on a parabolic fit in the axial MIP image. The dental arch including the teeth and the jawbone can be obtained by smoothly connecting the parabola representing the teeth and the parabola representing the jawbone as shown in Fig. 3. First, a teeth mask is acquired by applying a threshold to the axial MIP image (Fig. 3b). Then, after dividing the dental mask evenly in the angular direction with respect to the image center, the middle point of the masked teeth within each angular bin is marked as a fitting point. The RANSAC (RANdom Sample Consensus) algorithm was applied to the marked points to come up with the fitting parabola to the teeth^31^. The jawbone-fitting parabola was obtained by fitting a parabola connecting the marked points of the molar teeth and the tips of the corresponding jawbone (Fig. 3c). Finally, the jawbone-fitting parabola and the teeth-fitting parabola were merged by use of a cosine weighting function near the intersection points to smoothly connect them resulting in the dental arch delineation (Fig. 3d).

### Panoramic projection data acquisition

After acquiring the appropriate dental arch, the acquired dental arch is used to establish a panoramic scan trajectory. Again, it is noted that the virtual panoramic scan trajectory can be established by shifting the reference panoramic scan trajectory according to the patient’s dental arch. In this study, the manufacturer’s conventional panoramic scanning trajectory was used as the reference trajectory.

There are two major challenges in virtual panoramic imaging as proposed in this work: panoramic projection data synthesis from half-fan cone-beam projection data and intensity nonuniformity compensation over the panoramic FOV. In the following, we address these challenges and remedies one by one.

**Figure 4:**
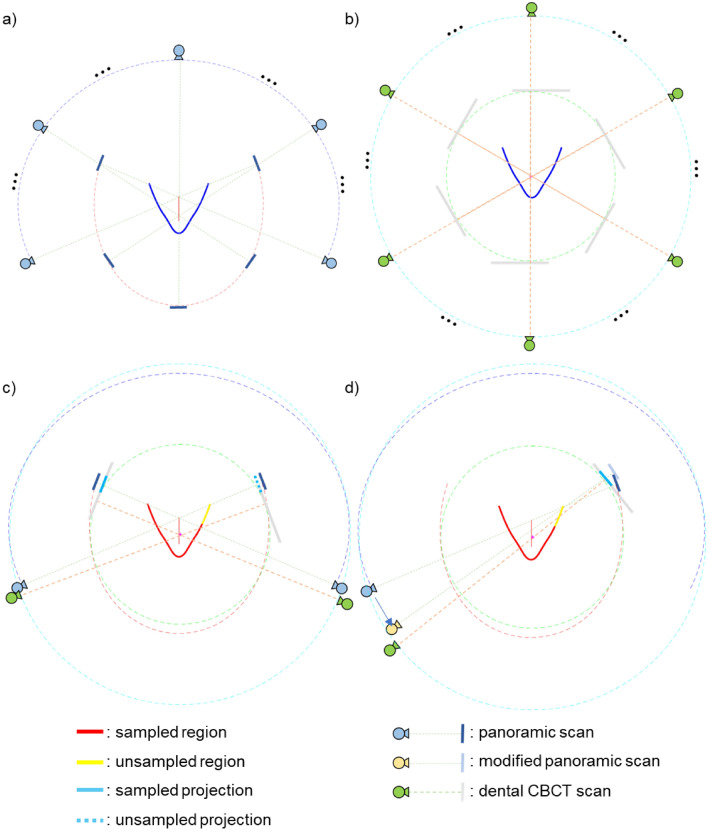
(**a**) Panoramic scan system. (**b**) Dental CBCT scan system. (**c**) Initial panoramic scan trajectory and sampling issue. (**d**) Modifying panoramic scan trajectory.

As shown in Fig. 4a and b, there are some differences between the panoramic scan system and the CBCT scan system. Unlike the panoramic scan system, the dental CBCT scan system uses a wide detector but uses a half-fan detector mode with an offset. In addition, while the rotational axis of the dental CBCT scan system is fixed, the rotational axis of the panoramic scan system moves along a line segment to minimize the overlapping of teeth images during a scan.

Due to the movement of the rotation axis in the panoramic scan system and the asymmetry of the detector fan-angle in the dental CBCT system, some projection data of the set panoramic scan trajectory cannot be obtained from the dental CBCT projection data as shown in Fig. 4c. The upper right tail part of the dental arch, represented by the yellow line segment, in this work would be subject to such data loss. The reason why specific panoramic projection data cannot be extracted from the dental CBCT projection data is that the beam path is too far from the fixed rotation axis of the dental CBCT system to be covered by the short fan-angle of the offset detector. In this work, we approximated those missing panoramic projection data by extending the virtual panoramic scan range so that the CBCT projection data that covers the yellow tail of the dental arch can be used for panoramic tomosynthesis as shown in Fig. 4d. Extending the virtual panoramic scan trajectory thus allows the beam path of the corresponding view to be closer to the rotation axis of the dental CBCT scan system.

**Figure 5:**
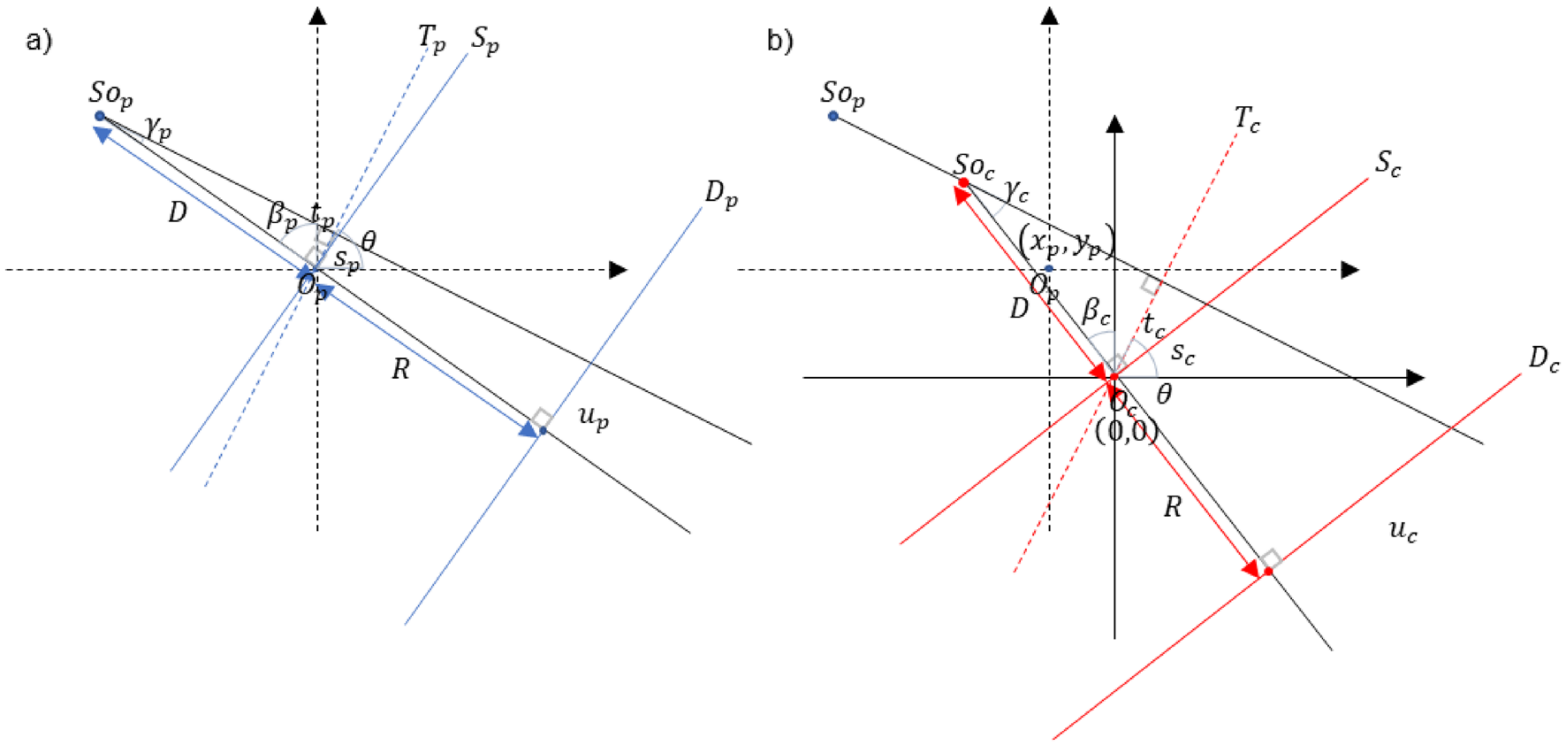
(**a**) Geometry of panoramic beam. (${\mathbf{S}\mathbf{o}}_{\mathbf{p}}$, Source position; ${\mathbf{D}}_{{\varvec{p}}}$, Detector position; ${\mathbf{O}}_{{\varvec{p}}}$, Rotation center), (**b**) Geometry of CBCT beam. (${\mathbf{S}\mathbf{o}}_{\mathbf{c}}$, Source position; ${\mathbf{D}}_{\mathbf{c}}$, Detector position; ${\mathbf{O}}_{\mathbf{c}}$, Rotation center).

After setting a virtual panoramic scan trajectory as described above, virtual panoramic projection data were extracted from the CBCT projection data. Since the source positions along a panoramic scan trajectory are not exactly overlapping those along a CBCT scan trajectory, ray matching and rebinning are necessary. The target panoramic beam was first transformed into a parallel beam through fan-parallel rebinning^32^. After that, the CBCT beam corresponding to the target panoramic beam was found by converting the parallel beam into a fan beam according to the CBCT system. Both panoramic beam and CBCT beam geometries are shown in Fig. 5. First, we convert the panoramic beam into the parallel beam as shown in Fig. 5a. So_p_ represents the source position of the panoramic scan, D_p_ detector plane, and *O*_p_ represents the rotation axis. ${\upbeta }_{\mathrm{p}}$ represents the panoramic beam source angle, ${\upgamma }_{\mathrm{p}}$ represents the fan-angle and $\uptheta$ represents the corresponding parallel beam angle. ${\mathrm{u}}_{\mathrm{p}}$ represents the panoramic beam detector coordinate, ${\mathrm{s}}_{\mathrm{p}}$ represents the virtual fan-beam detector coordinate, ${\mathrm{t}}_{\mathrm{p}}$ represents the radial parallel coordinate, D is the distance from the source to the rotation axis and R is the distance from the rotation axis to the detector surface. The relationship between $\left(\uptheta ,{\mathrm{t}}_{\mathrm{p}}\right)$ and $\left({\upbeta }_{\mathrm{p}},{\upgamma }_{\mathrm{p}},{\mathrm{s}}_{\mathrm{p}}\right)$ is given by Eq. (1).

1 $$\left\{\begin{array}{c}{\mathrm{s}}_{\mathrm{p}}={\mathrm{u}}_{\mathrm{p}}\frac{D}{D+R}\\ \genfrac{}{}{0pt}{}{{\upgamma }_{\mathrm{p}}=\mathrm{atan}\left({\mathrm{s}}_{\mathrm{p}}/\mathrm{D}\right)}{\uptheta ={\upgamma }_{\mathrm{p}}+{\upbeta }_{\mathrm{p}}}\\ {\mathrm{t}}_{\mathrm{p}}=\frac{D{s}_{p}}{\sqrt{{D}^{2}+{s}_{p}^{2}}}\end{array} \right.$$

As shown in Fig. 5b, the parallel beam is now converted again into a CBCT fan beam. ${\mathrm{So}}_{\mathrm{c}}$ represents the source position of the CBCT scan system, ${\mathrm{D}}_{\mathrm{c}}$ detector plane, and ${O}_{\mathrm{c}}$ represents the rotation axis. Unlike ${O}_{p}$, ${O}_{\mathrm{c}}$ has a fixed position. ${\upbeta }_{\mathrm{c}}$ represents the CBCT beam source angle, ${\upgamma }_{\mathrm{c}}$ represents the fan-angle, ${\mathrm{s}}_{\mathrm{c}}$ represents the virtual fan-beam detector coordinate, ${\mathrm{t}}_{c}$ represents the radial parallel coordinate. The relationship between $\left(\uptheta ,{\mathrm{t}}_{\mathrm{p}},{t}_{c}\right)$ and $\left({\upbeta }_{\mathrm{c}},{\upgamma }_{\mathrm{c}},{\mathrm{s}}_{\mathrm{c}}\right)$ is given by Eq. (2).

2 $$\left\{\begin{array}{c}{\mathrm{t}}_{\mathrm{c}}={\mathrm{t}}_{\mathrm{p}}+{\mathrm{x}}_{\mathrm{p}}cos\theta +{\mathrm{y}}_{\mathrm{p}}sin\theta \\ \genfrac{}{}{0pt}{}{{\mathrm{t}}_{\mathrm{c}}=\frac{D{s}_{c}}{\sqrt{{D}^{2}+{s}_{c}^{2}}},{\mathrm{s}}_{\mathrm{c}}=\frac{D{t}_{c}}{\sqrt{{D}^{2}-{t}_{c}^{2}}}}{{\mathrm{u}}_{\mathrm{c}}={\mathrm{s}}_{\mathrm{c}}\frac{D+R}{D}}\\ \genfrac{}{}{0pt}{}{{\gamma }_{c}=atan\left({s}_{c}/D\right)}{{\beta }_{c}=\theta -{\gamma }_{c}}\end{array}\right.$$

We can convert the panoramic beam $\left({\upbeta }_{\mathrm{p}},{\upgamma }_{\mathrm{p}},{\mathrm{s}}_{\mathrm{p}}\right)$ to the CBCT beam $\left({\upbeta }_{\mathrm{c}},{\upgamma }_{\mathrm{c}},{\mathrm{s}}_{\mathrm{c}}\right)$ using the two equation sets. In this way, panoramic projection data can be extracted from CBCT projection data along the virtual panoramic projection scan trajectory.

The second issue to be addressed is attenuation correction in panoramic projection data synthesis. In the projection rays that pass through the cervical vertebra in particular, the ray integrals of attenuation coefficients are much higher than those of projection rays that do not pass through the cervical vertebra. In the existing panoramic scan systems, the low-intensity projections caused by the cervical vertebrae are usually compensated by increasing the beam energy or effectively increasing the exposure in the relevant scanning range. However, in the case of panoramic projection data synthesis from CBCT projection data, the beam intensity in the raw projection domain should be appropriately corrected for by software.

**Figure 6:**
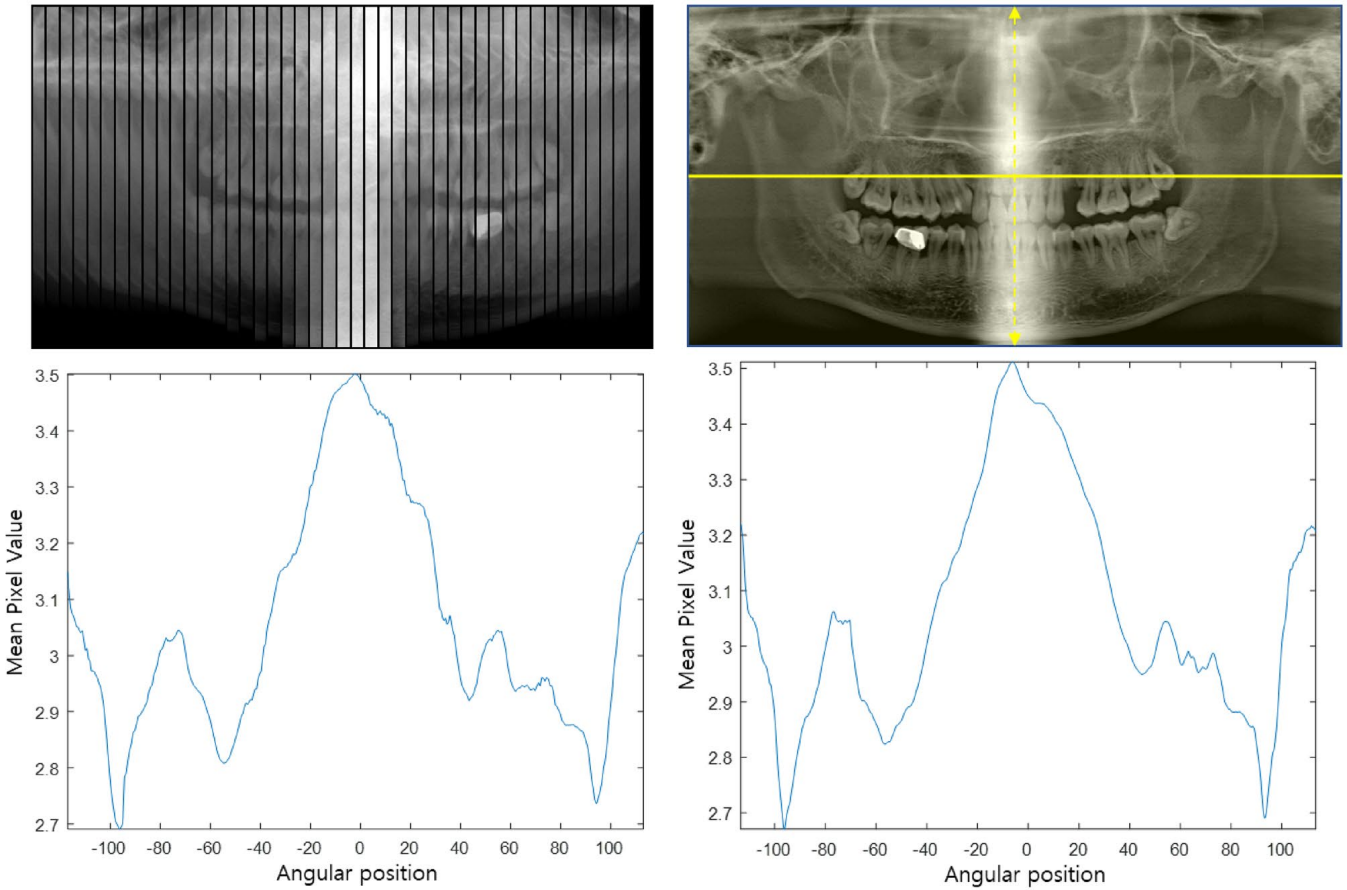
(Left) The mean pixel value of panoramic projection data according to angular position, (Right) the mean pixel value of panoramic image according to angular position.

As can be seen from Fig. 6, the synthesized panoramic projection data shows a substantial nonuniformity, and this nonuniformity directly translates to the reconstructed panoramic image. In this study, after calculating the average pixel value of the entire panoramic projection data (${\mathrm{m}}_{\mathrm{tot}}$) and the average pixel value of each projection data (${\mathrm{m}}_{\mathrm{proj}}$), an appropriate weight for each projection data is computed based on the ratio of the average pixel values (${\mathrm{w}}_{\mathrm{proj}}= {\mathrm{m}}_{\mathrm{tot}}/{\mathrm{m}}_{\mathrm{proj}}$). The view-by-view weighting factor thus acquired was then multiplied to all the pixels in the corresponding projection.

### Panoramic image reconstruction

A panoramic image is now reconstructed using the acquired panoramic projection data. In this paper, the tomosynthesis-based panoramic image reconstruction method, which is frequently used in the conventional panoramic scan system, was used. The tomosynthesis-based panoramic image reconstruction method uses the shift-and-add method with varying amounts of shift for producing multi-focal plane images^10,12^. The position of the focal plane on which the panoramic image is formed depends on the shift amount^10,12^. As the shift amount increases, a focal plane is generated farther from the source; and as the shift amount decreases, a focal plane is generated closer to the source. It is therefore possible to create a panoramic image of multiple slices with different depths of focus along the dental arch. To obtain a single panoramic image with the best focus, an auto-focusing method is applied that divides the sliced image into patches and finds and combines those with the highest sharpness. We would like to refer to our earlier publication for more details on the auto-focusing method^12^. Finally, the following edge enhancement algorithm was applied to increase the visibility of the acquired panoramic image^18^.

3 $$I= {\alpha }_{0}{I}_{0}+{\alpha }_{1}\left({I}_{0}-{G}_{1}\right)+{\alpha }_{2}\left({I}_{0}-{G}_{2}\right)+{\alpha }_{3}\left({I}_{0}-{G}_{3}\right)$$

, where ${\alpha }_{\mathrm{n}}$ denotes the weighting factors used to control the level of details and ${G}_{n}$ represents a 2D Gaussian filtered image of the original panoramic image ${I}_{0}$. The values of ${\alpha }_{\mathrm{n}}$ used in this work were empirically set by: ${\alpha }_{0}=1.0,{\alpha }_{1}=1.0,{\alpha }_{2}=1.5,{\alpha }_{3}=1.5$. The waists of the Gaussian filtering functions were: ${\upsigma }_{\mathrm{G}1}=2.4,{\upsigma }_{\mathrm{G}2}=4.8,{\upsigma }_{\mathrm{G}3}=19.2$. $I$ represents the final panoramic image.

### Data acquisition

Clinical dental CBCT projection data of 40 patients were retrospectively collected after de-identification under the institutional review board (IRB) approved all methods and the waived of informed consent by Well Dental Clinic in Teheran-ro, Seoul, Republic of Korea. All methods in this work were performed in accordance with the guidelines and regulations of Well Dental Clinic IRB. Also, a head phantom was scanned for a comparison study. The data sets were acquired using a rainbow CT (Dentium, Republic of Korea) scanner with 94 kVp, 8 mA (pulsed mode) parameters. The CBCT scanner used in this study is a 2-in-1 device with panoramic scanning capability. The scan parameters used in the CBCT scan system and the panoramic scan system are summarized in Table 1.

**Table 1:** Scan system parameters.

Parameter	CBCT scan	Panoramic scan
Distance of source to detector	609 mm	609 mm
Distance of source to isocenter	402 mm	402 mm
Detector array size	654 × 664	84 × 1328
Detector pixel size (mm)	0.24 × 0.24	0.12 × 0.12
Energy	98kvp	98kvp
Current	8mAs	8mAs
Image array size	512 × 512 × 350	2553 × 1328
Image pixel size (mm)	0.31	0.11
Rotation center	Fixed	Movable
Scan angle(degree)	360	230
Number of views	600	2553
Reconstruction method	FDK reconstruction	Shift-and-add

## Results

The proposed method was tested on a computer with the following specifications (Intel(R) Core(TM) i7-10700 K CPU 3.80 GHz, 64 GB RAM, NVIDIA GeForce RTX 2080 Ti, Windows 10). The method was implemented using the CUDA library for acceleration. The average running time required to acquire panoramic projection data was about 5.5 s, and the image reconstruction took about 6.2 s.

### Head phantom

**Figure 7:**
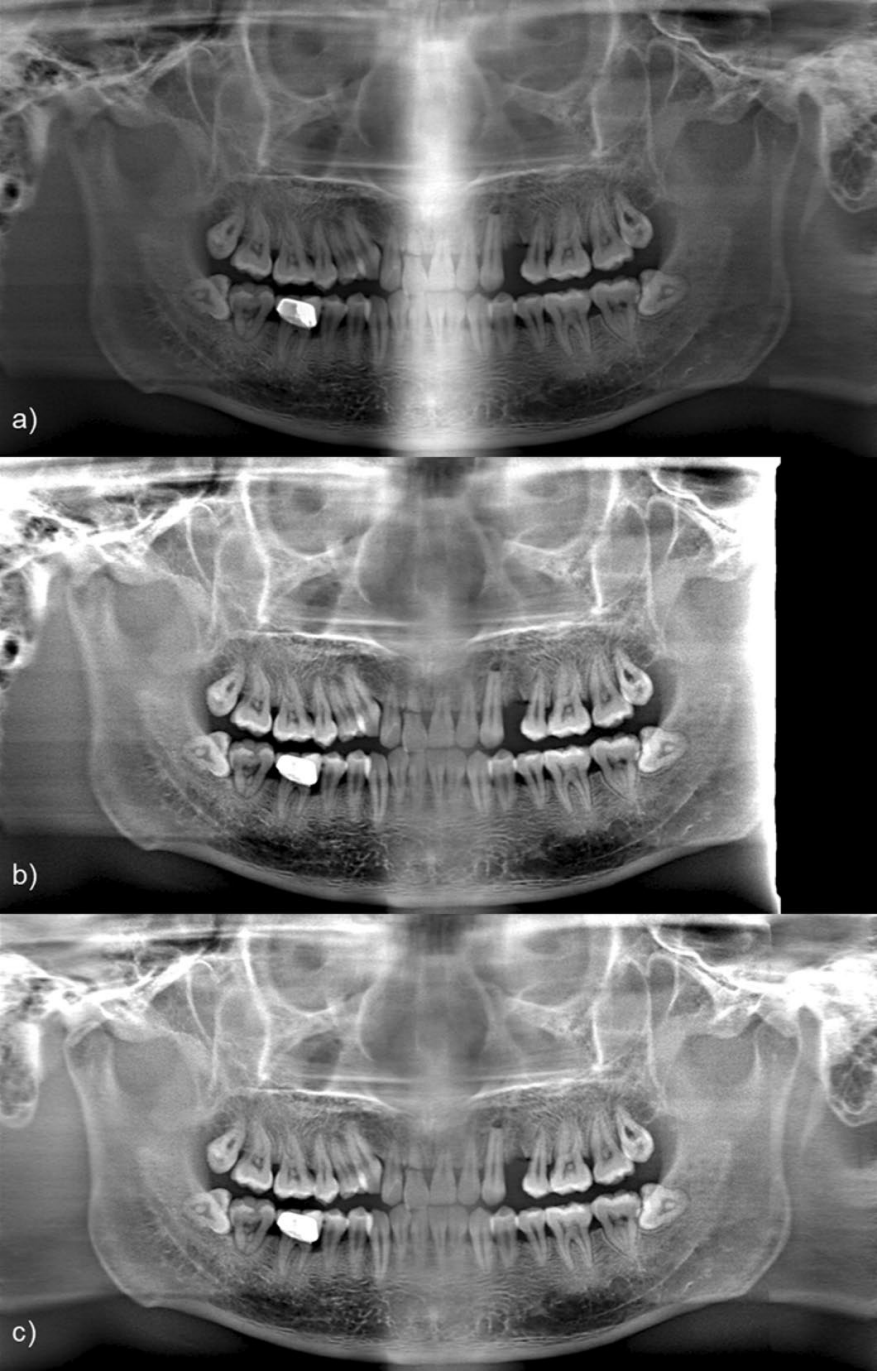
(**a**) A panoramic image without beam intensity correction, (**b**) A panoramic image without panoramic scan trajectory correction. (**c**) A panoramic image with both beam intensity and scan trajectory correction.

To check the effects of the panoramic scan trajectory correction and the beam intensity correction, panoramic images without each correction were acquired and compared in Fig. 7. In Fig. 7a, the panoramic image without beam intensity correction shows that the incisor area using projection data passing through the cervical vertebra has a significantly high intensity compared to the other areas. Unless the beam intensity is corrected, the overall visibility is reduced. In addition, as can be seen in the panoramic image without panoramic scan trajectory correction (Fig. 7b), when the panoramic scan trajectory is not corrected, some of the projection data is not sampled. Thus, the overall visibility is also compromised.

**Figure 8:**
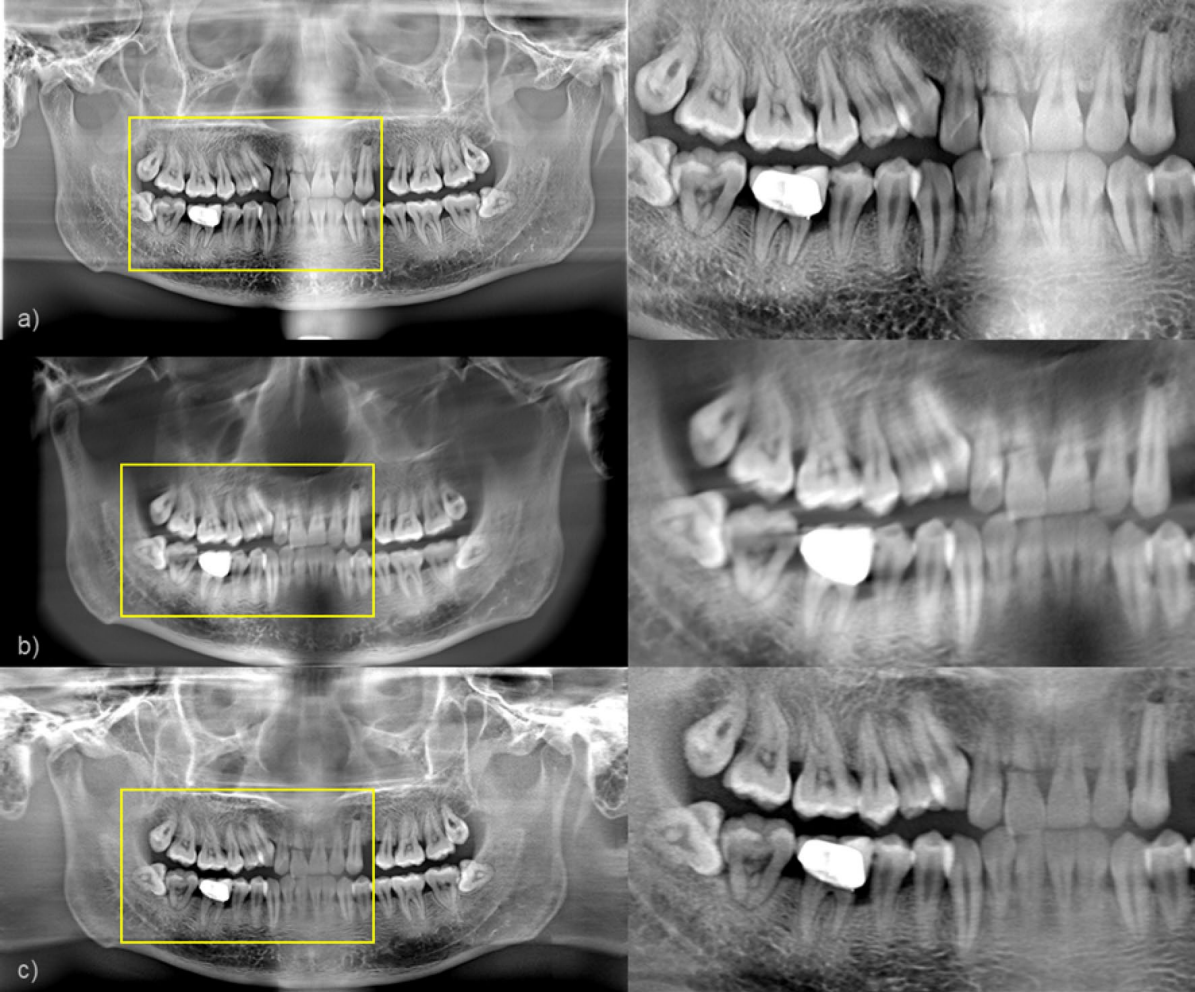
(**a**) A panoramic image obtained by the panoramic scan system, (**b**) A panoramic image synthesized from the dental CBCT image, and (**c**) A panoramic image obtained by the proposed method.

For image quality comparison, a panoramic image acquired with a panoramic scanning system (Fig. 8a) and a panoramic image synthesized from a CT image volume (Fig. 8b) by use of a conventional method, which is briefly described in the introduction part, were compared with a panoramic image acquired using the proposed method (Fig. 8c). Figure 8a is considered a gold standard.

The panoramic image obtained from the CBCT image (Fig. 8b) shows the overall teeth structure of the patient, but the resolution of the panoramic image is rather poor. In addition, it is observed that the metal artifact of the CBCT image remains in the panoramic image as streaky shades near the metal object. In Fig. 8c, it is confirmed that the resolution of the image reconstructed by the proposed method is higher than that of a panoramic image directly obtained from a CBCT image. CBCT image is composed of pixels with their size of 0.31 mm whereas the panoramic image is presented by pixels with their size of 0.11 mm. Since the proposed method makes use of CBCT original projection data of which detector pixel size is 0.24 mm, the resulting panoramic image carries higher spatial resolution. It is also noted that the spatial resolution of the real panoramic image is highest since the detector pixel size is smaller and the number of projection views is larger than those of CBCT, respectively.

### Clinical cases

**Figure 9:**
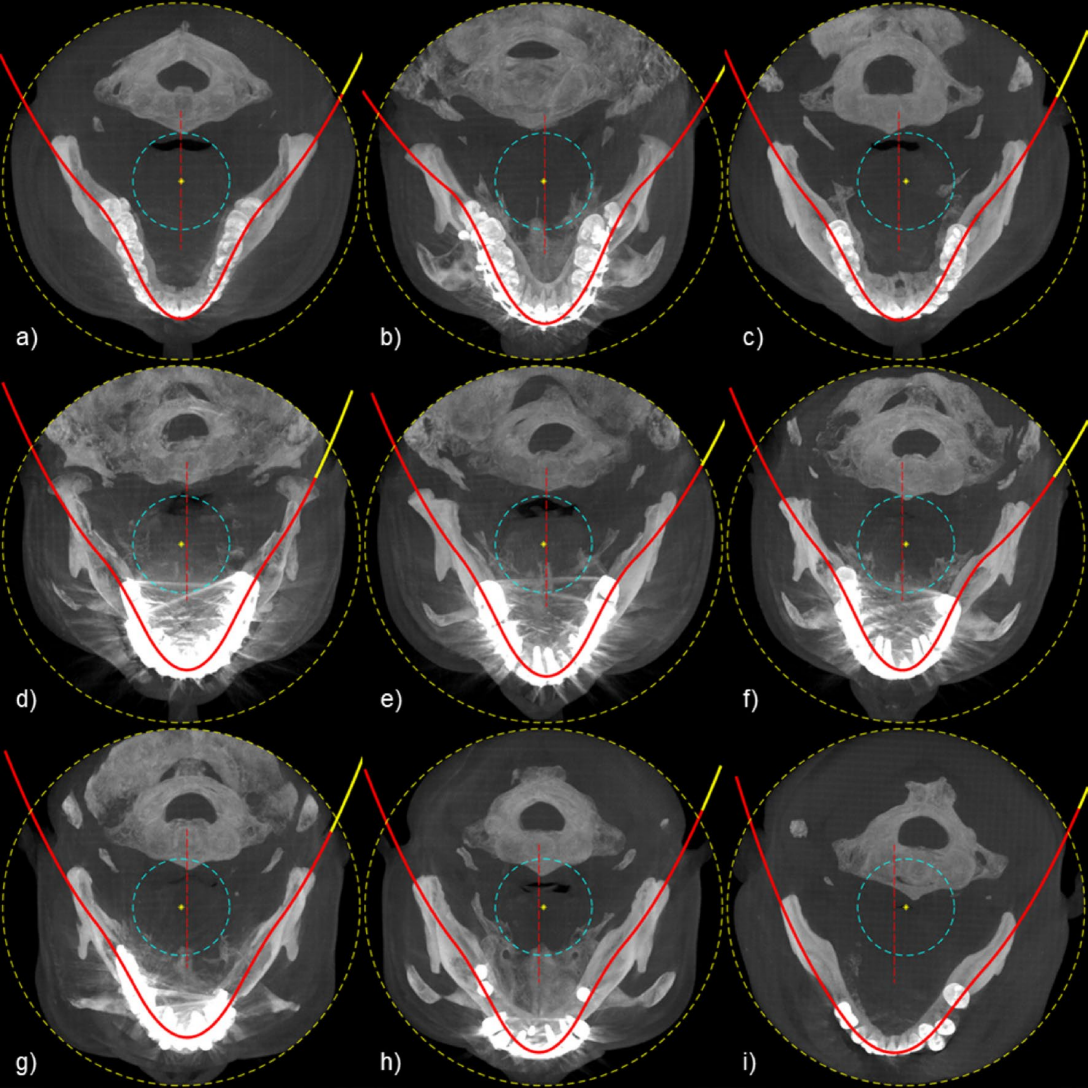
Dental arch and panoramic scan trajectory extracted from clinical case. (**a**–**c**) Cases where all teeth are present, (**d**–**f**) Cases with many metal prostheses, and (**g**–**i**) Cases of missing teeth.

The dental arch and the virtual panoramic scan axis extracted by the proposed method for various clinical cases are shown in Fig. 9. As shown in the figure, it is confirmed that the dental arch and the virtual panoramic scan axis are consistently formed not only when all the teeth are present (Fig. 9a–c) but also when there are severe metal artifacts (Fig. 9d–f) or missing teeth (Fig. 9g–i).

**Figure 10:**
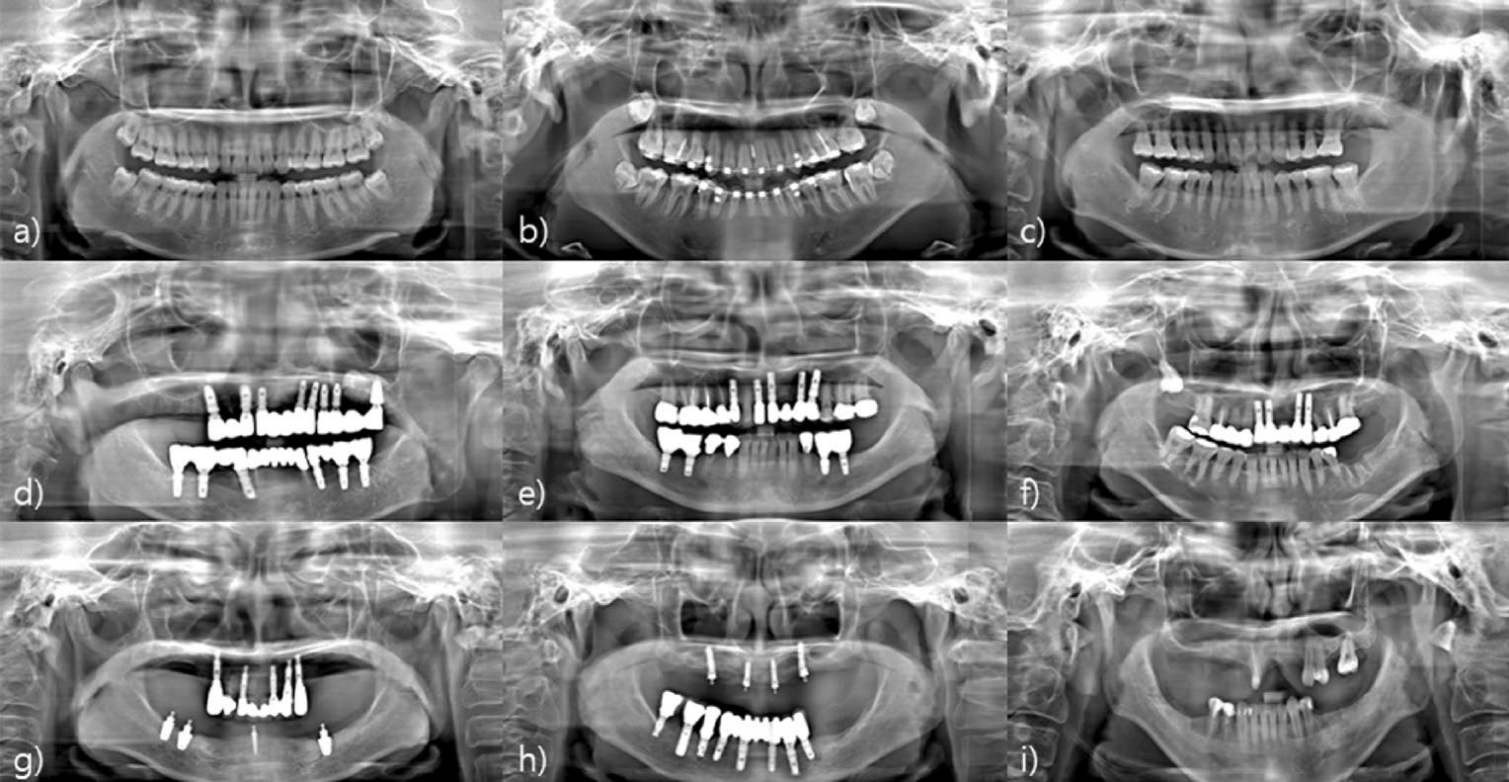
Panoramic images obtained using the proposed method. (**a**–**c**) Cases where all teeth are present, (**d**–**f**) Cases with many metal prostheses, and (**g**–**i**) Cases of missing teeth.

The panoramic images reconstructed by the proposed method for the clinical cases are shown in Fig. 10. Quality panoramic images were obtained from the clinical data of various cases. Even in cases with severe metal artifacts, panoramic images were successfully acquired based on the established panoramic scan trajectory. In addition, in the cases of many missing teeth, panoramic images naturally harmonize the part without teeth and the part with teeth.

**Figure 11:**
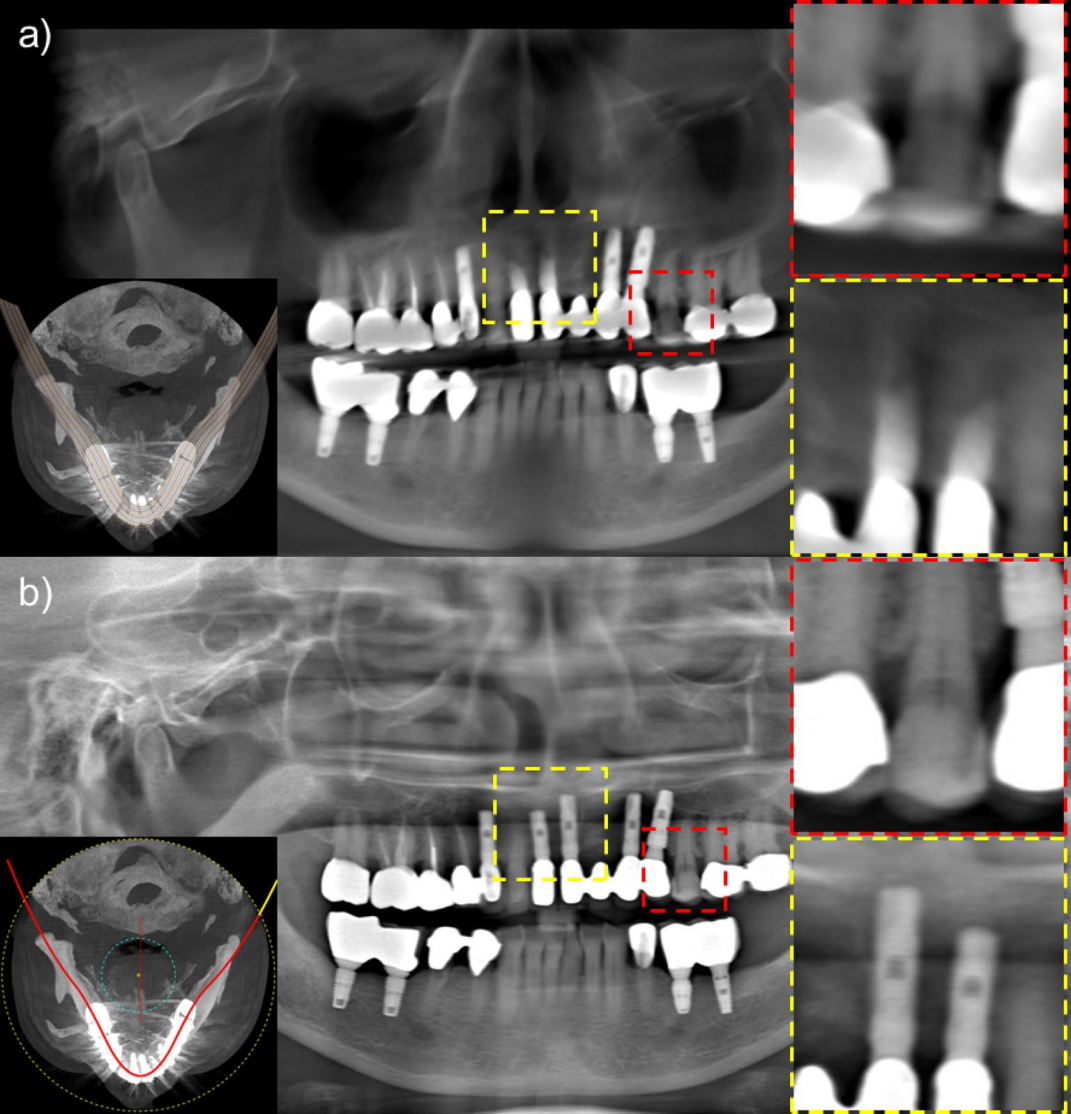
(**a**) A panoramic image synthesized from the dental CBCT image by use of a conventional method, (**b**) A panoramic image obtained by the proposed method.

To compare the proposed method with the CBCT-image-based method, or a conventional method, in the clinical case that has severe metal artifacts, we show the reconstructed images in Fig. 11. As shown in the red box in Fig. 11a, the teeth between the prosthesis may appear darker due to the beam hardening that contributes dominantly to metal image artifacts in CT. However, in the case of the proposed method, since narrow panoramic projection data are extracted and used, the effects of the prosthesis are much less. Zoomed-in images of the yellow box in Fig. 11 also reveal that the CBCT-image-based panoramic image reconstruction may lose substantial anatomy due to the poor image quality of CBCT with severe image artifacts.

## Discussion

The method of synthesizing panoramic images from dental CBCT images is highly subject not only to the image quality of CBCT but also to the accuracy of the dental arch. We have developed a reliable method of dental arch delineation that can be useful for both CBCT-image-based method and the proposed data synthesis method although the CBCT-image-based method still suffers from the image quality degradation due to image artifacts such as metal artifacts. It has been successfully demonstrated that the proposed panoramic image reconstruction method can robustly reconstruct the images in various clinical cases and that the proposed method outperforms the existing CBCT-image-based panoramic image synthesis. Particularly, advantages of the proposed method over the existing one in the cases with severe metal artifacts in CBCT images have been highlighted.

**Figure 12:**
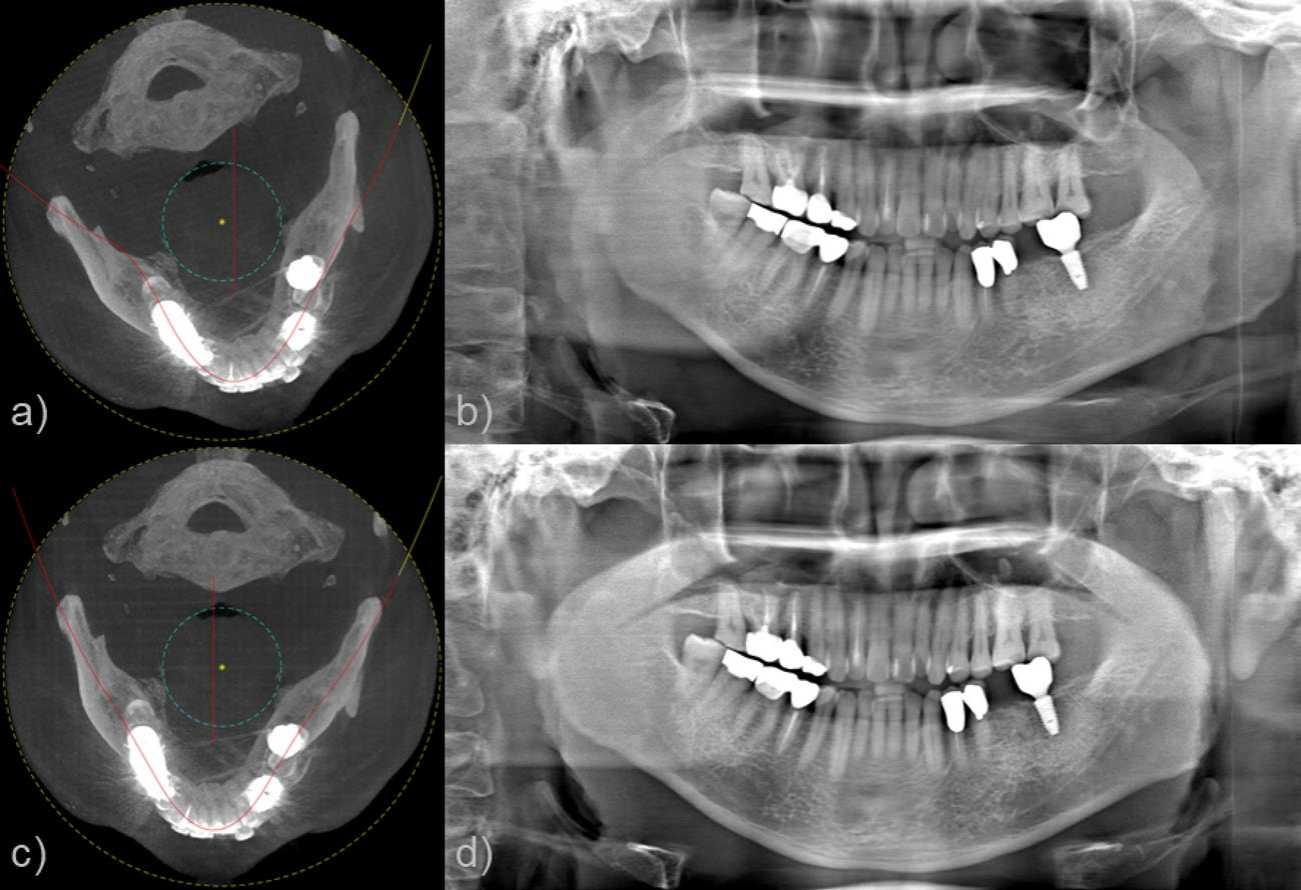
(**a**) The detected dental arch and panoramic scan trajectory when the patient's head is misaligned, (**b**) A panoramic image obtained by the proposed method. (**c**) Dental arch and panoramic scan trajectory extracted after head realignment, (**d**) A panoramic image obtained by the proposed method.

One of the strengths of the proposed method exists in selecting the dental arch and its associated virtual panoramic scan trajectory flexibly once the CBCT projection data are available. Figure 12 shows an example of demonstrating such flexibility. When the patient's head is positioned with an angular misalignment as shown in Fig. 12a, it may result in a tilted or distorted dental arch and accordingly a misaligned panoramic image as shown in Fig. 12b. One can rotate the original CBCT image and recalculates the dental arch and the virtual panoramic scan trajectory as shown in Fig. 12c. The panoramic image acquired from this modified virtual trajectory provides distortion-free image as shown in Fig. 12d. These days, automated artificial-intelligence driven approaches are actively investigated and deployed in various clinical image processing procedures. While we believe such innovations can also find applications in dental arch detection, virtual panoramic trajectory construction, and so on, we feel that comparison with such techniques in this article is beyond the scope.

We would like to note again that the proposed method is not aiming at replacing the panoramic imaging system used in the initial diagnosis, which is quite often practiced in clinics without taking a CBCT scan. In various clinical applications, after CBCT imaging occurs, our approach would render a more efficient way of synthesizing a virtual panoramic image than the CBCT-image-based panoramic image synthesis. We also note that the magnification effects, if there exists a difference of source-to-detector distance in the virtual panoramic system and the real panoramic scan system, may exist in the images produced by the proposed method compared to the real panoramic images.

## Conclusions

In this study, an image reconstruction method was proposed to obtain a virtual panoramic image from CBCT projection data instead of directly using a CBCT image. It is composed of dental arch delineation, setting up a virtual panoramic scan trajectory, recruiting virtual panoramic data from the CBCT projection, reconstructing the panoramic image slices, and compositing the final panoramic image by applying an auto-focusing technique. The proposed method successfully reconstructed panoramic images even in the presence of severe metal artifacts or missing teeth in CBCT images. The proposed method is believed to play an important role in various dental procedures that require panoramic images on top of CBCT images.

## Data Availability

The datasets generated and/or analyzed in this study are not publicly available due to the restrictions under license for the current study but are available from the corresponding author on reasonable request.
